# Mucinous carcinoma of the breast: epidemiological, clinical, and prognostic characteristics; a single-center experience

**DOI:** 10.1007/s12672-025-03146-2

**Published:** 2025-07-23

**Authors:** Omar Hamdy, Mosab Shetiwy, Mahmoud M. Saber, Basma A. Eldawody, Shorouq A. Kassab, Mariam H. Nabih, Mostafa Abdelhakiem, Mona Zaki, Shaimaa M. Yussif, Saleh Saleh, Khaled Abdelwahab

**Affiliations:** 1 Surgical Oncology Department, Oncology Center, Mansoura University, Mansoura, 35516 Egypt; 2 Faculty of Medicine, Mansoura University, Mansoura, Egypt; 3 Medical Oncology Unit, Oncology Center, Mansoura University, Mansoura, Egypt; 4 Radiology Department, Faculty of Medicine, Mansoura University, Mansoura, Egypt; 5 Pathology Department, Faculty of Medicine, Mansoura University, Mansoura, Egypt

**Keywords:** Mucinous carcinoma, Breast cancer, Prognosis, Survival

## Abstract

**Introduction:**

Mucinous (colloid) carcinoma (MC) of the breast typically affects postmenopausal and elderly women, with a more favorable prognosis compared to invasive breast carcinoma of no special type (IBC-NST). It is characterized by the presence of extracellular mucin and better outcomes. In our work, we presented a fifteen-year yield of a tertiary cancer center for MC.

**Methods:**

In this retrospective study, the data of the patients with MC from January 2009 to August 2023 were retrieved by searching the prospectively registered electronic database of the Oncology Center, Mansoura University. The patients’ epidemiological, clinical, pathological, therapeutic, and oncological data were analyzed.

**Results:**

A total of 152 patients with the pathology of MC of the breast were included. The mean age of patients was 55.38 ± 13.82 years. Imaging revealed a unifocal lesion in 93 patients (61.2%). The mean mass size by imaging was 37.25 ± 20.21 mm. Positive lymph nodes (LNs) were detected by imaging in 71 (46.7%) patients. Pathological variants were either pure MC (42.1%) or mixed mucinous ductal carcinoma (57.9%). Luminal A was the most common subtype. Neoadjuvant therapy (NAT) was received by 34.8% of the patients. Mastectomy was done for 103 patients (68.2%). Axillary lymph node dissection was done for 122 patients (80.3%), and sentinel lymph node biopsy (SLNB) was done for 30 patients (19.7%). Adjuvant chemotherapy and radiotherapy were received by 65.1% and 60.8% of patients, respectively, while adjuvant hormonal therapy was received by 84.5%. The mean disease-free survival (DFS) was 43 ± 34.02 months, while the mean overall survival (OAS) was 44.5 ± 33.46 months. Seventeen patients (11.2%) were reported dead during the follow-up period.

**Conclusion:**

MC of the breast is a unique type of breast cancer. It may mimic benign lesions on imaging. The primary treatment for MC is mostly surgery, followed by adjuvant radiotherapy and systemic therapy. Comparing MC to IBC-NST, the former had a better prognosis and fewer lymphatic metastases, especially with pure MC, which shows a better prognosis.

## Introduction

Globally, breast cancer is the most prevalent female cancer and the leading cause of cancer-related mortality [1]. The histological type of breast cancer is one of the important prognostic factors, helping to classify various subtypes of the disease and create more targeted treatments [2].

According to the last World Health Organization (WHO) classification, breast cancer can be divided into 21 different types based on different histopathological characteristics, of which IBC-NST is the most common type. Other special subtypes, including mucinous carcinoma, comprise approximately 25% of all breast cancer cases [2].

MC usually affects postmenopausal and elderly women, with a more favorable prognosis compared to IBC-NST [3]. It is characterized microscopically by the extracellular mucin (ECM) material that is separated by capillary-containing fibrous septa. The mucin, which is almost entirely extracellular, varies in extent and is separated by fibrous septae containing capillaries. The tumor cells are mildly atypical, uniform cells arranged in small clusters as solid, acinar, or micropapillary structures [4].

In this manuscript, we present a fifteen-year yield of a tertiary cancer center of MC. We analyzed the epidemiological, diagnostic, therapeutic, and prognostic characteristics to provide a spotlight on this tumor category.

## Methods

### Source

In this retrospective study, the data of the MC patients from January 2009 to August 2023 were retrieved by searching the prospectively registered electronic database of the Oncology Center, Mansoura University. The patients’ epidemiological, clinical, pathological, therapeutic, and oncological data were analyzed.

### Variables

For each patient who was included, the following information was gathered:

- Epidemiological: medical comorbidities, age, and body mass index (BMI).
- Clinical: mass location, size, focality, lymph node (LN) status, BIRADS, and clinical stage.
- Pathological: preoperative biopsy, pathological type, hormone receptor (HR), and human epidermal growth factor receptor 2 (HER2) status, ki67 (defined as low when less than 20% and high when equal to or more than 20%), postoperative pathology and luminal subtype (Luminal A: HR + HER2-low ki67, Luminal B: HR + HER2-high ki67 or HR + HER2+, HER2 enriched: HR-HER2+, triple negative: HR-HER2-. If Ki67 is not available, for earlier cases, it was clarified that HR + HER2- were considered luminal A-like if they showed low to intermediate histologic grade, low mitotic count, and high PR expression, while HR + HER2 + or HR + HER2- tumors with high grade, high mitotic count or low PR were considered Luminal B–like biology). HER2 was considered positive if score 3 by IHC or score 2 but had positive amplification by in situ hybridization (ISH), and it was considered negative for scores 0, 1, and score 2 but had negative amplification by ISH [5].
- Management: neoadjuvant treatment, breast surgery type, axillary staging type, adjuvant treatment.
- Prognosis: locoregional recurrence, metastasis, and survival.

### Inclusion criteria

The patients’ data were included if they received surgical management and if the final pathologic diagnosis was:

- Mucinous carcinoma.
- Mixed mucinous carcinoma.
- Carcinoma with mucinous component.

### Exclusion criteria

- Unregistered or missed data.
- No surgical intervention.
- Unsure diagnosis.

### Outcomes

The objectives of the study are the assessment of disease epidemiology, pathological and management data, as well as the incidence of recurrence and distant metastasis. The primary endpoint was disease-free survival, while locoregional recurrence and distant metastasis were the secondary endpoints.

### Statistical analysis

The data of patients in this study were analyzed using Statistical Package for the Social Sciences (SPSS) version 26 (Inc., Chicago, IL) on MacOS. Continuous variables were presented as means or medians and ranges (according to their distribution), while categorical variables were presented as proportions. Bivariate analysis was done using the Chi-Square test. Independent sample T-test was used to compare the means of different groups and dependent variables. A *p-value* of < 0.05 was considered statistically significant. DFS, defined as the time from diagnosis until relapse or progression, and OAS were computed using the Kaplan–Meier method, and the significance of survival differences among selected variables was verified using the log-rank test.

## Results

A total of 152 MC patients were included. Descriptive values for age, BMI, co-morbidities, presence of palpable mass, imaging type and size of the lesion, presence of multifocal/multicentric lesions, imaging status of LNs, imaging density of breast tissue, BIRADs scoring of lesions, clinical TNM staging, preoperative biopsy type and pathology, biological markers, neoadjuvant therapy, and its response, type of surgery performed, postoperative pathology and adjuvant therapies, recurrence site (local and distant) and survival are listed in Tables 1 and 2.

**Table 1:** Numerical values of the patient characteristics

	Number of patients with available data	Minimum	Maximum	Mean	SD
Age	152	20	91	55.38	13.82
BMI	120	22.2	55	35.69	6.74
Imaging size (mm)	149	3.5	130	37.25	20.21
Pathological size (mm)	145	5	240	41.56	30.85
No of retrieved LNs	148	0	43	13.52	7.7
No of positive LNs	148	0	23	2.79	4.5
DFS	152	1	172	43*	34.02
OAS	152	2	172	44.5*	33.46

*LNs* lymph nodes, *BMI* body mass index, *SD* standard deviation

*For DFS (disease-free survival) and OAS (overall survival) the reported value is the median, not the mean

**Table 2:** Categorial values of the patient characteristics

Variable		Number (%)
*Comorbidities*		
Diabetes Mellitus	No	110 (72.4)
	Yes	42 (27.6)
	Total	152 (100)
Hypertension	No	96 (63.2)
	Yes	56 (36.8)
	Total	152 (100)
Cardiac	No	130 (85.5)
	Yes	22 (14.5)
	Total	152 (100)
Hepatic	No	140 (92.1)
	Yes	12 (7.9)
	Total	152 (100)
Others	Polio	2 (8)
	Bronchial asthma	7 (28)
	Mentally retarded	1 (4)
	Gastroesophageal reflux disease	1 (4)
	hyperthyroidism	1 (4)
	Cataract	1 (4)
	thyroidectomy	1 (4)
	peripheral neuropathy	1 (4)
	osteoarthritis	1 (4)
	cerebral stroke	2 (8)
	hypothyroidism	1 (4)
	rheumatoid arthritis	2 (8)
	Alzheimer’s disease	1 (4)
	retinal detachment	2 (8)
	Psychosis	1 (4)
	Total	25 (100)
*Clinical examination*		
Palpable mass	No	8 (5.3)
	Yes	144 (94.7)
	Total	152 (100)
*Imaging*		
Ultrasound	No	3 (2)
	Yes	149 (98)
	Total	152 (100)
Mammography	No	10 (6.6)
	Yes	142 (93.4)
	Total	152 (100)
Magnetic resonance imaging (MRI)	No	134 (88.2)
	Yes	18 (11.8)
	Total	152 (100)
Mass	No	3 (2)
	Yes	149 (98)
	Total	152 (100)
Site*	UOQ	74 (48.7)
	UIQ	26 (17.1)
	LOQ	20 (13.2)
	LIQ	25 (16.4)
	Central	7 (4.6)
	Total	152 (100)
Focality	Unifocal	93 (61.2)
	Multifocal	35 (23)
	Multicentric	24 (15.8)
	Total	152 (100)
Lymph node status	Negative	59 (38.8)
	Equivocal	22 (14.5)
	Positive	71 (46.7)
	Total	152 (100)
Breast density (ACR)	A	23 (20.2)
	B	60 (52.6)
	C	21 (18.4)
	D	10 (8.8)
	Total	114 (100)
BIRADS	2	2 (1.5)
	3	7 (5.1)
	4a	16 (11.8)
	4b	19 (14)
	4c	29 (21.3)
	5	63 (46.3)
	Total	136 (100)
T Stage (tumor size according to TNM)	1	20 (13.4)
	2	90 (60.4)
	3	33 (22.1)
	4	6 (4)
	Total	149 (100)
*Preoperative pathological data*		
Biopsy type	Core needle biopsy	134 (88.7)
	FNAC	5 (3.3)
	Excisional	12 (7.9)
	Total	151 (100)
Pathological type	Mucinous	64 (42.1)
	Mucinous + ductal	88 (57.9)
	Total	152 (100)
Estrogen receptor (ER)	Negative	9 (7.8)
	Positive	106 (92.2)
	Total	115 (100)
Progesterone receptor (PR)	Negative	18 (15.9)
	Positive	95 (84.1)
	Total	113 (100)
Her2neu	Negative	75 (67)
	Positive	37 (33)
	Total	112 (100)
Ki67	Low	27 (33.8)
	High	53 (66.3)
	Total	80 (100)
Luminal Type	Luminal A	56 (48.3)
	Luminal B	52 (44.8)
	Triple-negative	5 (4.3)
	Her2 enriched	3 (2.6)
	Total	116 (100)
*Preoperative treatment*		
Neoadjuvant therapy (NAT)	No	99 (65.1)
	Yes	53 (34.8)
	Total	152 (100)
NAT response	No response	7 (13.2)
	Partial response	34 (64.2)
	Complete response	9 (17)
	N/A	3 (5.6)
	Total	53 (100)
*Surgery*		
Surgery type	Mastectomy	103 (67.8)
	Breast conservative surgery	44 (28.9)
	Reconstruction	5 (3.3)
	Total	152 (100)
Surgery axillary staging	SLNB	30 (19.7)
	Axillary dissection	122 (80.3)
	Total	152 (100)
Pathological LN status	Negative	67 (44.1)
	Positive	85 (55.9)
	Total	152 (100)
*Postoperative pathology*		
Grade	Grade I	6 (7.7)
	Grade II	65 (83.3)
	Grade III	7 (9)
	Total	78 (100)
Lymphovascular invasion	No	88 (63.3)
	Yes	51 (36.7)
	Total	139 (100)
*Postoperative treatment*		
Adjuvant chemotherapy	No	53 (34.8)
	Yes	99 (65.1)
	Total	152 (100)
Adjuvant hormonal therapy	No	23 (15.5)
	Yes	125 (84.5)
	Total	148 (100)
Adjuvant radiotherapy	No	58 (39.2)
	Yes	90 (60.8)
	Total	148 (100)
*Prognosis*		
Recurrence	No	130 (85.5)
	Yes	22 (14.5)
	Total	152 (100)
Recurrence, local site	Breast	4 (66.7)
	Axilla	2 (33.3)
	Total	6 (100)
Recurrence, distant site	Lung	3 (15.8)
	Brain	2 (10.5)
	Bone	6 (31.6)
	Liver	1 (5.3)
	Multiorgan	6 (31.6)
	Ovary	1 (5.3)
	Total	19 (100)
Last follow up status	Alive	135 (88.8)
	Dead	17 (11.2)
	Total	152 (100)

*In multicentric tumors, the mentioned site is for the index tumor only

The mean age of the included patients was 55.38 ± 13.82 years. The mean BMI was 35.69 ± 6.74 kg/m^2^. Clinically, a mass was palpable in 144 of the included 152 patients (94.7%), and the LNs were enlarged in 40% of patients.

**Fig. 1:**
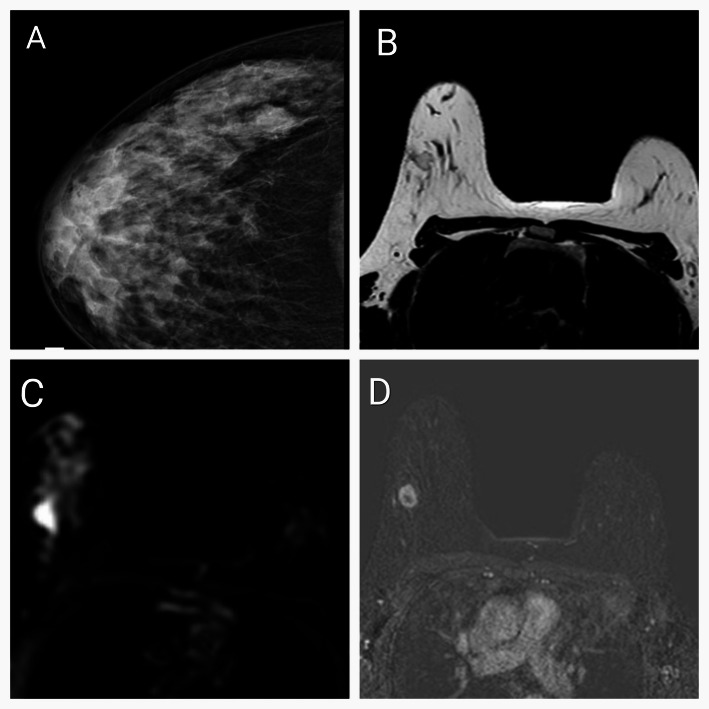
Imaging of mucinous breast carcinoma: **A** CC mammogram view shows an indistinct margin, high-density lesion in the right upper outer quadrant. **B** MRI, T2WI, the lesion shows high signal intensity (SI). **C** MRI, diffusion-weighted image (DWI), the lesion shows high SI on high b value. **D** MRI, Early subtraction image shows rapid heterogeneous enhancement of the lesion

Regarding imaging (Fig. 1), most of the patients in the study had an ultrasound (US) examination or a sonomammography (98% and 93.4%, respectively), while only 18 patients underwent magnetic resonance imaging (MRI) (11.8%). Only three patients had no detected masses by imaging (2%). Almost half of the tumors were in the upper outer quadrant (74 patients, 48.7%). Imaging revealed a unifocal lesion in two-thirds (93) of the patients (61.2%), while multifocal and multicentric diseases were detected in 35 patients (23%), and 24 patients (15.8%), respectively. The mean mass size by imaging was 37.25 ± 20.21 mm. Positive LNs were detected by imaging in 71 (46.7%) patients. 63 patients had a BIRADs V lesion (46.3%).

**Fig. 2:**
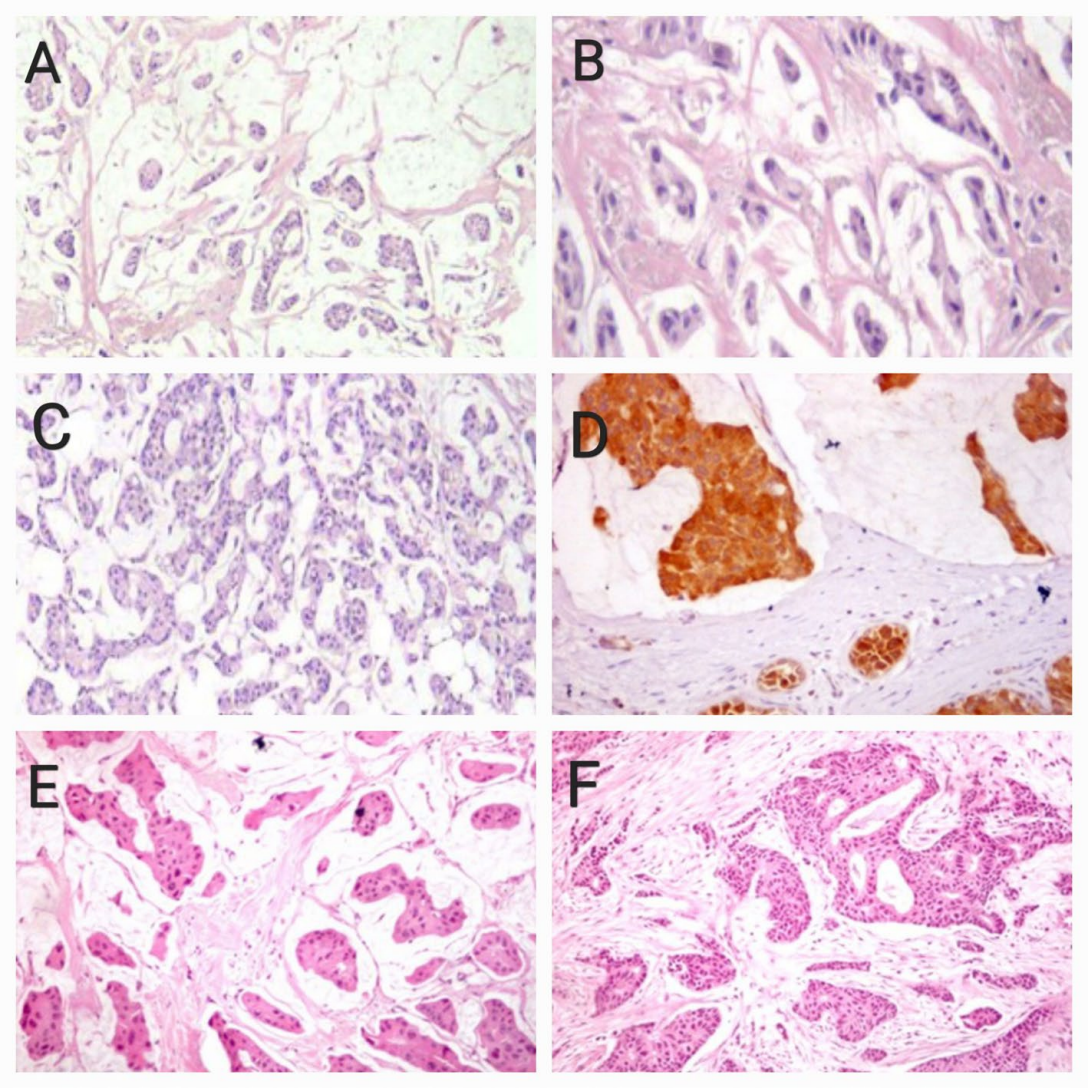
Microscopic examination of mucinous breast carcinoma: **A** A case of PMC, type A, shows pools of mucin > 90% of the tumor with floating small clusters of tumor cells (Hx & E x 100). **B** Type A tumor cells form small clusters, strands, or acini (Hx & E x 400). **C** A case of PMC, type B, shows large sheets of tumor cells with mucin and neuroendocrine differentiation (Hx & E x 200). **D** Type B mucinous carcinoma with neuroendocrine differentiation shows a positive cytoplasmic reaction for synaptophysin (IHC x 400). **E** A case of MMC showing pools of mucin > 50% of the tumor with floating aggregates of tumor cells forming acinar structures (Hx & E x200). **F** MMC showing areas of invasive breast carcinoma of no special type (IBC-NST) < 50% of the tumor mass (Hx & E X200)

The main method of preoperative biopsy was core needle biopsy (88.7%). Unplanned resections were encountered in only 12 (7.9%) of patients. Pathological variants (Fig. 2) were either pure mucinous (42.1%) or mixed mucinous ductal carcinoma (57.9%). Luminal A & Luminal B were the most common subtypes (48.3% and 44.8%, respectively).

Neoadjuvant therapy was received by 34.8% of the patients. Mastectomy was done for 103 patients (68.2%) while breast-conserving therapy was done for 28.5% of patients, and only five patients underwent breast reconstructive surgery. Axillary lymph node dissection was done for 122 patients (80.3%), and sentinel lymph node biopsy (SLNB) was done for 30 patients (19.7%).

Positive LNs were reported in 55.9% of patients. The mean number of retrieved LNs was 14 ± 8. The mean number of positive LNs was 3. The mean pathological size of the lesion was 41.6 ± 30.9 mm.

Adjuvant chemotherapy and radiotherapy were received by 65.1% and 60.8% of patients, respectively, while adjuvant hormonal therapy was received by 84.5%.

**Fig. 3:**
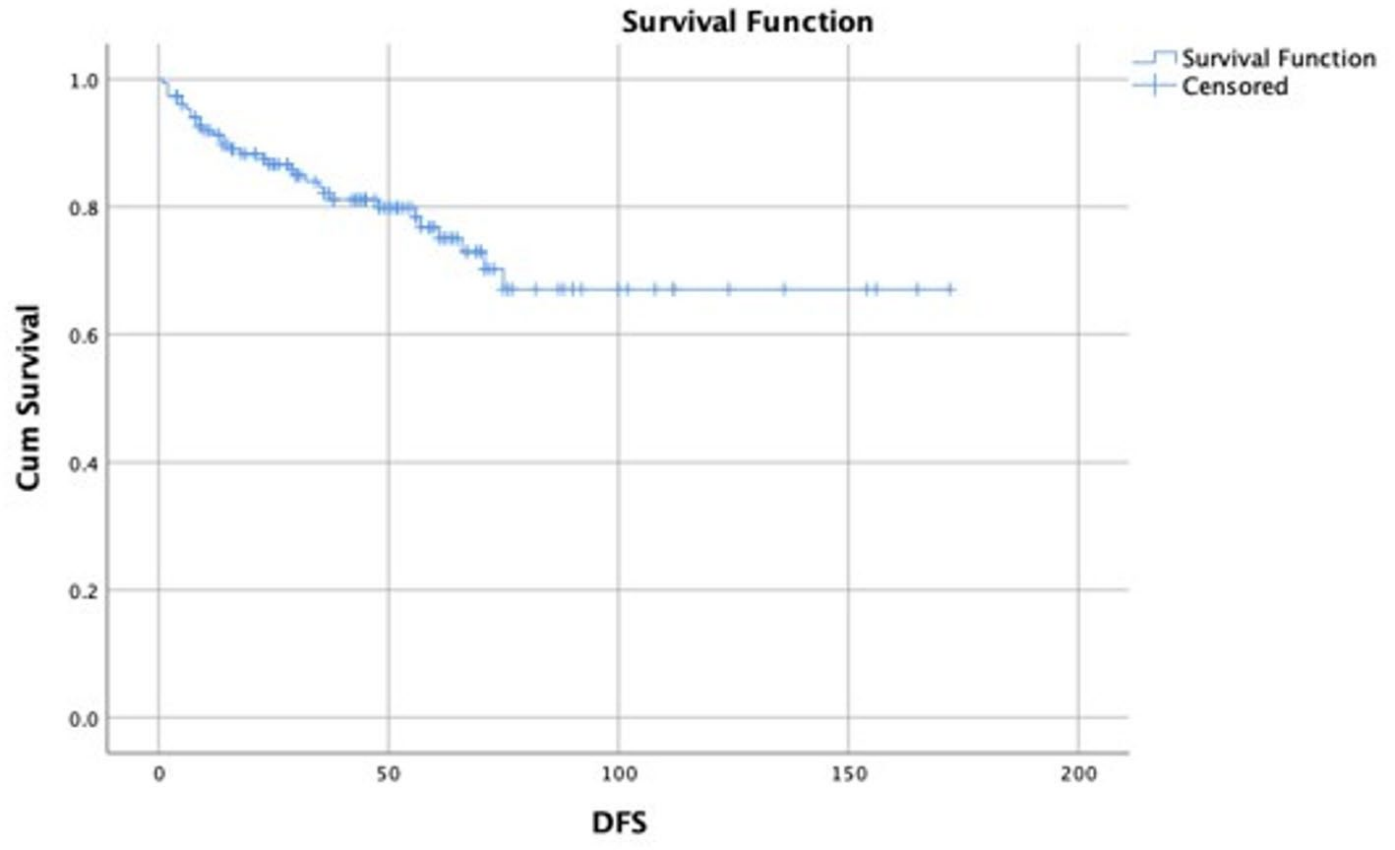
A Kaplan Maier curve showing the DFS of the included patients

**Fig. 4:**
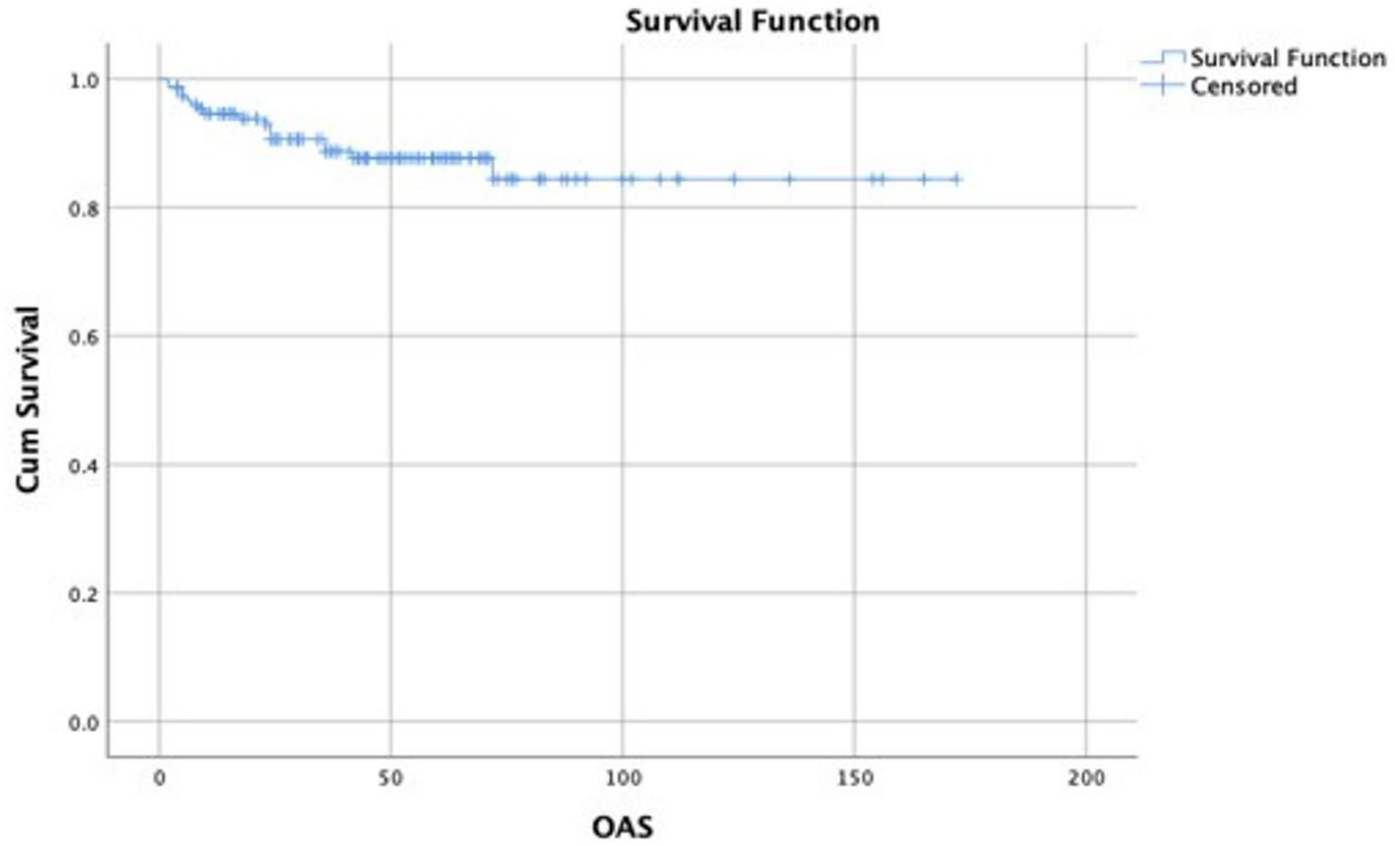
A Kaplan-Meier curve showing the OAS of the included patients

Recurrence occurred in 22 patients (14.5%). The mean DFS was 43 ± 34.02 months while the mean OAS was 44.5 ± 33.46 months. Seventeen patients (11.2%) were reported dead during the follow-up period (Figs. 3 and 4).

**Fig. 5:**
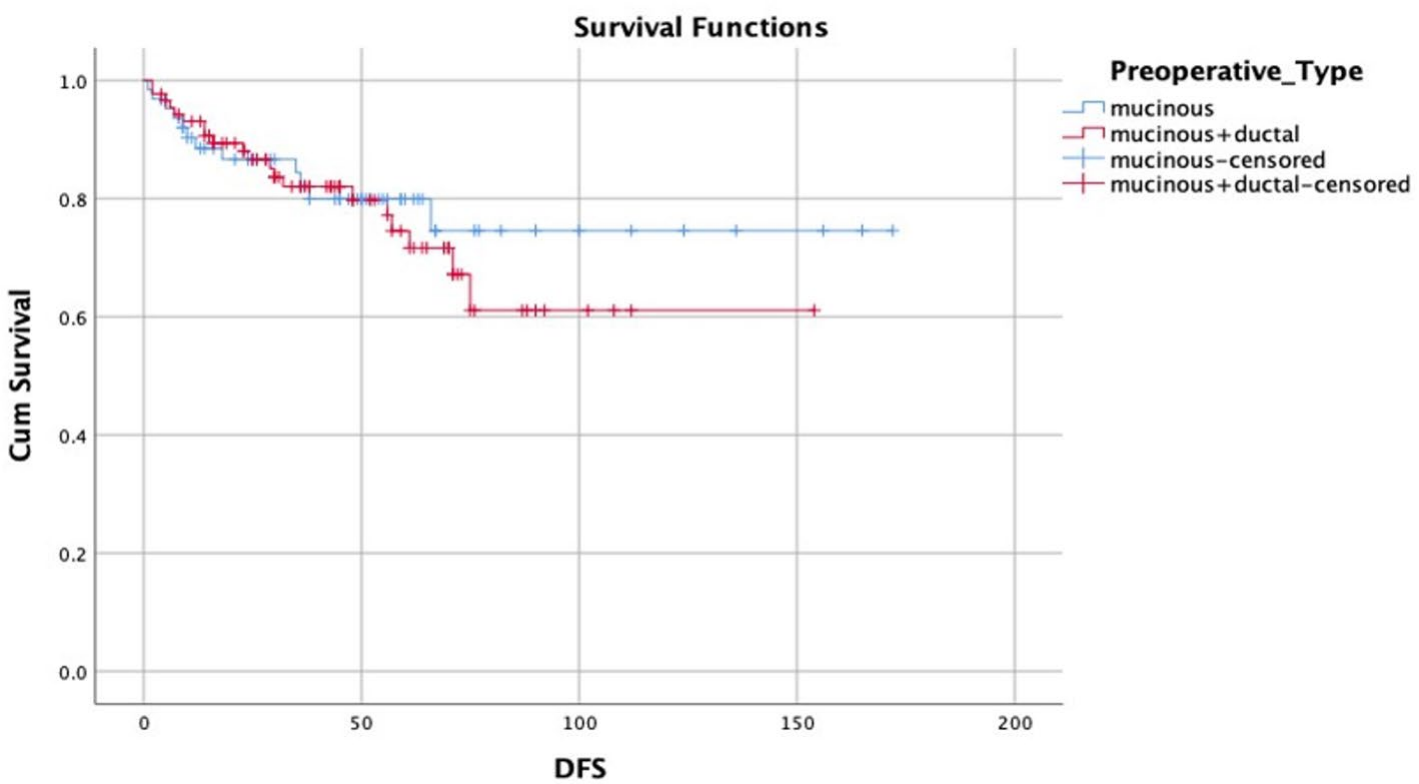
A Kaplan-Meier curve showing the DFS of PMC vs. MMC

Correlation between the two different pathological types of mucinous breast cancer, the mixed and the pure types, showed statistical significance regarding the number of positive LNs (*p* < 0.001) (Table 3). The mixed pathology group reported a higher proliferation index (*p* = 0.001). Luminal B, HER2-enriched, and triple-negative breast cancer (TNBC) were reported more in the mixed pathology group (*p* = 0.05). The neoadjuvant response was detected more in the mixed pathology group (*p* = 0.005). Mixed pathology was linked more with frequent bony and multiorgan distant recurrences when compared to the pure mucinous type (*p* = 0.05) (Table 4, Fig. 5).

**Table 3:** Comparison between the two pathological types regarding the numerical variables

	Pathology	*N*	Mean	SD	SE mean	*P* value
Age	Mucinous	64	57.2	14.14	1.77	0.17
	Mucinous + ductal	88	54.05	13.52	1.44	
BMI	Mucinous	46	35.44	6.33	0.93	0.75
	Mucinous + ductal	74	35.84	7.02	0.82	
Size of the lesion (Imaging - mm)	Mucinous	62	37.76	21.56	2.74	0.79
	Mucinous + ductal	87	36.88	19.31	2.07	
Size of the lesion (Pathological - mm)	Mucinous	61	43.08	31.74	4.06	0.61
	Mucinous + ductal	84	40.45	30.34	3.31	
Number of positive LNs	Mucinous	62	1.27	2.91	0.37	**< 0.001**
	Mucinous + ductal	86	3.88	5.1	0.55	
DFS	Mucinous	64	48.44	39.88	4.99	0.45
	Mucinous + ductal	88	44.18	29.15	3.11	
OAS	Mucinous	64	50.17	39.39	4.92	0.52
	Mucinous + ductal	88	46.58	28.54	3.04	

*BMI* body mass index, *LNs* lymph nodes, *DFS* disease-free survival, *OAS* overall survival

Bold numbers for significant p=values

**Table 4:** Comparison between pure mucinous type vs. mixed mucinous + ductal

Variable		Mucinous	Mucinous + ductal	*P* value
Palpable mass	No	3 (2)	5 (3.3)	0.79
	Yes	61 (40.1)	83 (54.6)	
	Total	64 (42.1)	88 (57.9)	
Imaging mass	No	2 (1.3)	1 (0.7)	0.57
	Yes	62 (40.8)	87 (57.2)	
	Total	64 (42.1)	88 (57.9)	
Imaging site	UOQ	34 (22.4)	40 (26.3)	0.5
	UIQ	13 (8.6)	13 (8.6)	
	LOQ	6 (3.9)	14 (9.2)	
	LIQ	8 (5.3)	17 (11.2)	
	Central	3 (2)	4 (2.6)	
	Total	64 (42.1)	88 (57.9)	
Imaging MF MC	Unifocal	44 (28.9)	49 (32.2)	0.22
	Multifocal	13 (8.6)	22 (14.5)	
	Multicentric	7 (4.6)	17 (11.2)	
	Total	64 (42.1)	88 (57.9)	
Imaging LN status	Negative	31 (20.4)	28 (18.4)	**0.001**
	Equivocal	14 (9.2)	8 (5.3)	
	Positive	19 (12.5)	52 (34.2)	
	Total	64 (42.1)	88 (57.9)	
Imaging density ACR	A	10 (8.8)	13 (11.4)	0.24
	B	26 (22.8)	34 (29.8)	
	C	4 (3.5)	17 (14.9)	
	D	4 (3.5)	6 (5.3)	
	Total	44 (38.6)	70 (61.4)	
Imaging BIRADs	2	0	2 (1.5)	0.06
	3	4 (2.9)	3 (2.2)	
	4a	8 (5.9)	8 (5.9)	
	4b	12 (8.8)	7 (5.1)	
	4c	13 (9.6)	16 (11.8)	
	5	18 (13.2)	45 (33.1)	
	Total	55 (40.4)	81 (59.6)	
Clinical T stage	T1	11 (7.4)	9 (6)	0.33
	T2	39 (26.2)	51 (34.2)	
	T3	10 (6.7)	23 (15.4)	
	T4	3 (2)	3 (2)	
	Total	63 (42.3)	86 (57.7)	
ER	Negative	1 (0.9)	8 (7)	0.08
	Positive	43 (37.4)	63 (54.8)	
	Total	44 (38.3)	71 (61.7)	
PR	Negative	4 (3.5)	14 (12.4)	0.13
	Positive	39 (34.5)	56 (49.6)	
	Total	43 (38.1)	70 (61.9)	
Her2Neu	Negative	34 (30.4)	41 (36.6)	**0.02**
	Positive	8 (7.1)	29 (25.9)	
	Total	42 (37.5)	70 (62.5)	
Ki67	Low	17 (21.3)	10 (12.5)	**0.001**
	High	13 (16.3)	40 (50)	
	Total	30 (37.5)	50 (62.5)	
Luminal type	Luminal A	29 (25)	27 (23.3)	**0.05**
	Luminal B	15 (12.9)	37 (31.9)	
	Triple-negative	1 (0.9)	4 (3.4)	
	Her2 enriched	0	3 (2.6)	
	Total	45 (38.8)	71 (61.2)	
Neoadjuvant therapy (NAT)	No	42 (27.6)	57 (37.5)	0.8
	Yes	22 (14.5)	31 (20.3)	
	Total	64 (42.1)	88 (57.9)	
NAT response	No response	7 (13.2)	0 (0)	**0.005**
	Partial response	13 (24.5)	21 (39.6)	
	Complete response	1 (1.9)	8 (15.1)	
	N/A	1 (1.9)	2 (3.7)	
	Total	22 (42.3)	31 (59.6)	
Surgery type	Mastectomy	45 (29.6)	58 (38.1)	0.77
	Breast conservative surgery	17 (11.2)	27 (17.8)	
	Reconstruction	2 (1.3)	3 (2)	
	Total	64 (42.1)	88 (57.9)	
Pathological LN status	Negative	42(27.6)	25 (16.4)	**< 0.001**
	Positive	22 (14.5)	63 (41.4)	
	Total	64 (42.1)	88 (57.9)	
Pathology grade	Grade I	2 (2.6)	4 (5.1)	0.7
	Grade II	14 (17.9)	51 (65.4)	
	Grade III	1 (1.3)	6 (7.7)	
	Total	17 (21.8)	61 (78.2)	
Pathology LVI	No	43 (30.9)	45 (32.4)	**0.001**
	Yes	11 (7.9)	40 (28.8)	
	Total	54 (38.8)	85 (61.2)	
Recurrence	No	58 (38.2)	72 (47.4)	0.13
	Yes	6 (3.9)	16 (10.5)	
	Total	64 (42.1)	88 (57.9)	
Recurrence local site	Breast	1 (16.7)	3 (50)	0.44
	Axilla	0	2 (33.3)	
	Total	1 (16.7)	5 (83.3)	
Recurrence distant site	Lung	1 (5.3)	2 (10.5)	**0.05**
	Brain	2 (10.5)	0	
	Bone	1 (5.3)	5 (26.3)	
	Liver	0	1 (5.3)	
	Ovary	1 (5.3)	0	
	Multiorgan	0	6 (31.6)	
	Total	5 (26.3)	14 (73.7)	
Status	Alive	56 (36.8)	79 (52)	0.66
	Dead	8 (5.3)	9 (5.9)	
	Total	64 (42.1)	88 (57.9)	

Bold numbers for significant

When comparing the outcomes between the patients with positive (infiltrated) and negative LNs, the following was noted with statistical significance (Table 5). Younger patients were more likely to have positive LNs (*p* = 0.005). Clinical and radiological assessment of axillary LNs correlated with the postoperative pathological status of LNs (*p* < 0.001). Multifocal and multicentric breast tumors were associated with positive axillary LNs (*p* < 0.001). Disturbed breast cancer cases were less likely to have positive LNs (*p* < 0.001). MC was more likely to recur if the LNs were positive (*p* = 0.03). However, DFS and OAS were not statistically significant between the groups.

**Table 5:** Correlation between the nodal status and outcomes

Variable		Postoperative LN status		P value
		Negative	Positive	
Imaging LN	Negative	40 (26.3)	19 (12.5)	< 0.001
	Equivocal	15 (9.9)	7 (4.6)	
	Positive	12 (7.9)	59 (38.8)	
	Total	67 (44.1)	85 (55.9)	
Imaging MF/MC	Unifocal	53 (34.9)	40 (26.3)	< 0.001
	Multifocal	12 (7.9)	23 (15.1)	
	Multicentric	2 (1.3)	22 (14.5)	
	Total	67 (44.1)	85 (55.9)	
Disturbed	No	85 (56.3)	54 (35.8)	0.005
	Yes	12 (7.9)	0	
	Total	97 (64.2)	54 (35.8)	
Clinical T stage	T1	8 (5.3)	12 (8.1)	0.04
	T2	48 (32.2)	42 (28.2)	
	T3	9 (6)	24 (16.1)	
	T4	1 (0.7)	5 (3.3)	
	Total	66 (44.3)	83 (55.7)	
Luminal type	Luminal A	29 (25)	27 (23.3)	0.002
	Luminal B	11 (9.5)	41 (35.3)	
	TNBC	4 (3.4)	1 (0.9)	
	Her2 enriched	1 (0.9)	2 (1.8)	
	Total	45	71	
Neoadjuvant therapy (NAT)	No	55 (36.2)	44 (28.9)	< 0.001
	Yes	12 (7.9)	41 (27)	
	Total	67 (44.1)	85 (55.9)	
Pathology	Pure mucinous	42 (27.6)	22 (14.5)	< 0.001
	Mixed	25 (16.4)	63 (41.4)	
	Total	67 (44.1)	85 (55.9)	

*LN* lymph node, *MF* multifocal, *MC* multicentric

## Discussion

Pure MC (PMC) is classified into Capella type A and Capella type B according to the amount of ECM and cellularity [6]. Type A is hypocellular with abundant extracellular mucin and floating tumor cells either as small clusters, epithelial strands, or cribriform structures. Type B is hypercellular with large sheets of tumor cells with mucin production and neuroendocrine differentiation [6, 7]. Another variant of mucinous carcinoma is called mucinous micropapillary carcinoma (pure mucinous carcinoma with a micropapillary pattern). These tumors are characterized by moderate to high-grade nuclear features, a hobnail pattern, micropapillary pattern, and more frequent positive HER2 status [7]. In our cohort, PMC constituted 42.1% of the included patients while mixed mucinous carcinoma (MMC) constituted 57.9%.

PMC consists of over 90% mucinous content, typically present as stage I and II, luminal subtypes [6], with negative HER2 expression, and lower incidence of LN metastasis compared to IBC-NST [3]. It also has a reduced likelihood of metastasis. On the other hand, MMC contains mucinous content ranging from 50 to 90%, along with a solid component composed of IBC-NST, lobular, or neuroendocrine elements. The distinct clinicopathological parameters result in prognostic differences between PMC and MMC [6]. In our study, all the MMCs were composed of mucinous carcinoma and IBC-NST. They showed a higher tendency for more positive LNs. A higher proliferation index was reported in the mixed pathology group. Luminal B, HER2-enriched and TNBC were reported more in the mixed pathology group. In addition, adjuvant treatments (chemotherapy and radiotherapy) were reported to be received by patients in the mixed pathology group. Mixed pathology was also associated with more frequent bony and multiorgan distant recurrences.

In the diagnostic stage, MC may mimic a benign lesion on ultrasound. Typically, it appears as an oval or round mass, showing iso or hypo-echogenicity [8], and usually displaying poorly defined or micro-lobulated margins [7]. Additionally, it exhibits posterior acoustic enhancement and internal echoes, with cystic or solid components. PMC often presents with more frequent heterogeneous internal echoes compared to MMC [9]. Differentiating MC from benign lesions such as benign phyllodes tumors and fibroadenomas can be difficult due to various reasons. However, specific MRI features, such as the higher apparent diffusion coefficient (ADC) and the enhancing internal septations, can aid in making this distinction [8]. The lower tendency for LN metastasis also plays a role in the misdiagnosis of benign-looking lesions.

At mammography, PMC is usually presented as an oval or round mass showing iso or hyper-density and circumscribed or microlobulated margins. While MMC is usually presented with indistinct or speculated margins [9]. Approximately 20% of mucinous carcinomas cannot be detected mammographically or are presented as calcifications or focal asymmetries [8]. In our cohort, 25 patients (18.4%) were diagnosed as BIRADS 2,3, or 4a lesions by preoperative diagnostic imaging.

There is still a debate about the treatment of MC and other special types due to the lack of evidence and the rarity of cases, as most of the data comes from case reports and small series [8, 10]. Nevertheless, the primary treatment for MC is mostly surgery, either breast-conserving surgery or a mastectomy [11]. Furthermore, neoadjuvant therapy can lead to a pathological complete response (pCR) rate of 17.9% in MC, which helps enable breast-conserving surgery in these patients [12]. Yet, since our series included patients over a decade and a half, which witnessed a change in surgical practice from more to fewer mastectomies, it still shows that most patients underwent mastectomy & axillary dissection followed by breast-conserving surgery and SLNB.

Following surgical treatment, patients undergo adjuvant therapy as radiotherapy, chemotherapy, hormonal therapy, and targeted therapy. Compared to other breast cancer types, radiotherapy and chemotherapy are usually used at lower rates in MC patients; otherwise, hormonal therapy is more commonly used, as this tumor is often ER/PR positive, while targeted therapy is less commonly used [13]. MMC cases undergo mastectomy and need more frequent adjuvant chemotherapy treatments compared to PMC due to nodal metastasis [8]. This was the same observation in our study, as more patients with MMC received adjuvant chemoradiotherapy than PMC.

The 5-year overall survival of MC was found to be 94%, which is better than IBC-NST, which has a rate of 82% [14]. Nodal status is the most important prognostic factor, while age, tumor size, PR status, and grade come next [8]. In our cohort, the mean DFS was 45.97 ± 34.02 months while the mean OAS was 48.09 ± 33.46 months.

Comparing MC to IBC-NST, the former had a better prognosis and fewer LN metastases. Metastases from axillary LNs happen in 12–14% of patients [15]. Compared to MMC, PMC has a better prognosis. 81–94% remain disease-free after five years if no LN metastasis exists. PMC may show a late distant spread [16]. This was the same as reported in our study. In addition, mixed pathology was associated with more frequent bony and multiorgan distant recurrences than the pure mucinous type.

Our study has limitations, being retrospective, which led to selection bias. Also, there is heterogeneity in the surgical and medical treatment protocols during the large study period. In addition, some missed data could have affected the accuracy of our results. Lastly, the advances in imaging tools, digital mammography, high-resolution US, and the availability of MRI may have impacted the outcomes. However, the strengths can outweigh the limitations, such as presenting a tertiary referral center experience. Also, the number of patients included, and the study period are not small. Having a prospectively maintained database limited the selection bias.

## Conclusions

Mucinous carcinoma of the breast is a unique form of breast cancer that may be misdiagnosed as benign lesions on imaging. It is mostly treated by surgery, followed by adjuvant radiation and systemic therapy. When compared to IBC-NST, pure mucinous carcinoma had a better prognosis and fewer lymphatic metastases. Also, the pure mucinous type exhibits less aggressive behavior and a better prognosis than mixed mucinous carcinoma.

## Data Availability

All the clinical, radiological and pathological data used in this manuscript are available in the Mansoura University medical system (Ibn Sina Hospital management system) http://srv137.mans.edu.eg/mus/newSystem/.
